# Postpartum depression during COVID-19

**DOI:** 10.18332/ejm/122386

**Published:** 2020-05-15

**Authors:** Maria E. Almasidou

**Affiliations:** 1 Midwifery Department, University of West Attica, Athens, Greece; 2 Hippokration General Hospital, Thessaloniki, Greece

**Keywords:** midwives, COVID-19, virtual visits, teleconferencing, postpartum depression, EPDS

**Dear Editor,**

During the COVID-19 pandemic crisis midwives need to be more aware about postpartum depression. Many women who had already planned their childbirth and their life, face now extreme fear and stress. The fear and other health problems^1^ that mothers and infants develop usually appear in the first weeks of labor, including postpartum hemorrhage, fever, infection, abdominal and back pain, and also urinary tract complications that can lead to postpartum depression. Midwives have to be concerned, if postpartum depression was a common problem in one in nine of the women before the pandemic, now it can affect many more women. Women generally want to discuss^2^ their childbirth experience, preferably with the midwife who was present at birth.

In order to comply with social distancing^3^, midwives have to accomplish a psychometric examination via virtual visits with open camera. The midwife at the beginning and during the visit-teleconference should ask the woman questions^4^ related to her current psychological condition. Some of them are:

1. During the last month, did you feel psychologically depressed?
2. During the last month, have you had any interest in doing something?
3. In the last two weeks, how often have you felt nervous or anxious?

If some of the questions are affirmative, she is at risk of developing a psychological instability^4^.

Psychometric examination should be continued using a special EPDS questionnaire^5^. The questionnaire can be completed by the midwife by asking the questions or sending it to the woman to fill in at the time of the conference call, to avoid any interference from other family members. If the results of the answers are worrying, then the woman should be referred to a healthcare professional^4,6^. The midwife must recognize the seriousness of the situation in case of avoidance to respond by the woman or negligence to complete the questionnaire.

Women need to be encouraged to take care of themselves. This includes gentle exercise, time to rest, seeking help in caring for the baby, talking to someone about their feelings, and making sure they can have access to a virtual-midwife^4,6^ at any moment.

In virtual home visits it is not necessary to make many visits, as this will have negative effects such as developing postpartum depression1, but the midwife should provide more psycho-education via the teleconference^7^.

Teleconferencing should be based on empathy (i.e. the fight against negative emotions and loneliness) that helps mothers in their daily lives. In addition, it should be individualized and adapted^8,9^ to each situation and woman.
